# Sexual Addiction, Hypersexual Behavior and Relative Psychological Dynamics during the Period of Social Distancing and Stay-at-Home Policies Due to COVID-19

**DOI:** 10.3390/ijerph19052704

**Published:** 2022-02-25

**Authors:** Pasquale Caponnetto, Marilena Maglia, Graziella Chiara Prezzavento, Concetta Pirrone

**Affiliations:** 1 Department of Educational Sciences, University of Catania, 95121 Catania, Italy; p.caponnetto@unict.it (P.C.); khyaramely@msn.com (G.C.P.); concetta.pirrone@unict.it (C.P.); 2 Center of Excellence for the Acceleration of Harm Reduction (COEHAR), University of Catania, 95121 Catania, Italy; 3 CTA—Villa Chiara Psychiatric Rehabilitation Clinic and Research, 95030 Mascalucia, Italy

**Keywords:** Sexual Addiction, hypersexual behavior, SAST, COVID-19, lock-down

## Abstract

During the COVID-19 pandemic, increased stress factors affected people’s motivations to engage in potentially addictive behaviors. Sexuality, understood as one of the psychological aspects to be investigated to explore the level of psychological well-being of a person, has suffered considerable repercussions due to the pandemic. A growing body of evidence suggests an unprecedented increase in Internet use and online pornography consumption during the pandemic. Since March 2020, during the lockdown period, Pornhub has seen a worldwide increase in pornography use of 11.6% compared to the previous average days. This research was conducted with the aim of exploring the possible increasing use of pornographic material during the lockdown period, in order to assess whether dysfunctional behaviors, such as compulsive behaviors, and thoughts of sex-related obsessives can lead to hypersexual behavior or a more severe Sexual Addiction. The individuals who participated in our research were 18 years of age or older (mean 23.1, s.d. 5.8), and 48% male and 52% females and were recruited online to complete a self-report questionnaire in the period between April 2020 and April 2021. The questionnaires were delivered via main social networks. The tool used for our survey was the SAST (Sexual Addiction Screening Test), a questionnaire including socio-demographic data and data relating to sexual practices, such as sexual orientation and time spent on the Internet for sexual activities. The results revealed significant differences concerning the various factors investigated such as loss of control, addictive symptoms and hide score.

## 1. Introduction

During the COVID-19 pandemic, the increase in stressors (psychological, social, financial, occupational) impacted people’s motivations to engage in addictive behaviors [1]. Due to social distancing and stay-at-home policies, these behaviors were implemented using technological tools and the Internet. Negative emotional responses have been found in both the general adult population and in healthcare professionals, as well as in children and adolescents following the announcement of the COVID-19 pandemic. This is consistent with previous research that has found that such health-related public emergencies usually cause a series of stressful emotional responses characterized by high levels of anxiety and emotions, typically negative, along with a favorable feeling structure [2,3]. To better understand how the trauma linked to the pandemic and the resulting lockdown affected mental health, not only in the short term but also in a long term, several longitudinal studies were performed. A study conducted by Hyland et al. [4], in which the authors attempted to assess whether there were increases in cases of major depression and generalized anxiety disorder (GAD) between February 2019 and March-April 2020 and whether there were changes in the frequency of major depression and in the GAD during the six-week national lockdown, found no significant changes in depression and GAD during the lockdown. Therefore, these findings showed that the outbreak of the COVID-19 pandemic did not lead to an increase in major depression or GAD in general. Another study carried out in Italy by Gori and Topino [5] aimed to analyze trends in perceived stress, post-traumatic symptoms and state anxiety from March 2020 to March 2021. The results showed a significant decrease in post-traumatic symptoms from the first year of the COVID-19 pandemic with levels of perceived stress, worry and state anxiety that remained constant. Correlations between variables at different times were also explored, as well as gender differences throughout the year. Post-traumatic symptoms significantly decreased after the first phase of March 2020. This may be linked to a relaxation of restrictive measures, for which previous studies have highlighted a significant psychological impact. The highest scores were in fact, recorded at an early-stage limitation. Furthermore, this expands on Wang’s preliminary longitudinal study, which identified a statistically significant decrease in post-traumatic symptoms after four weeks in Chinese participants [6].

In recent years we have been witnessing a growing number of people who manifest new forms of addiction no longer related to the intake of psychoactive substances but related to the recurrence of behaviors that cause mental discomfort and suffering. Behavioral addictions are defined as “new addictions” to distinguish them from those involving the use of drugs and psychoactive substances. Pathological gambling, compulsive shopping, Internet addiction, work alcoholism, study addictions, physical activity and Sexual Addiction are part of the new addictions [7]. Currently, the fifth edition of the Diagnostic and Statistical Manual of Mental Disorders contains significant changes related to the diagnosing of addictive problems. These changes have included only the gambling disorder among the substance-related and addictive disorders and classified gambling as non-substance-related disorders [8]. All other addictions without substance are instead the subject of research, given their growing popularity. Sexual Addiction is a disorder characterized by intrusive and obsessive sexual thoughts and fantasies, associated with loss of control over sexual behavior that causes negative consequences at work, emotional, and social levels [7,9]. Defined sex addiction as the consequence of a hyper-sexuality of the subject, understood as an above-average sexual behavior. Liggio, identifying sex addiction as an addiction to endogenous drugs, proposes the term “addiction to orgasmic reaction” (DRO). This psychophysiopathological condition that is correlated with an alteration of the cerebral gratification system involving a coarctation of the modality in which the individual obtains satisfaction and pleasure [10]. All these definitions highlight an extremely significant aspect that manifests itself in subjects suffering from this disorder, the phenomenon of sexual craving. It is understood as an intense and uncontrollable desire that alters the state of consciousness of the subject to the point of making them lose control over their impulses. Just as happens in drug addicts, even individuals with Sexual Addiction undergo the phenomena of tolerance, abstinence and craving [11] where they will therefore feel the need to increase sexual behaviors or their intensity in order to maintain the desired effect and will subsequently manifest real psychophysiological changes, such as an increase in anxious symptoms [12]. The hypothesis that Hypersexual Disorder could be a psychiatric disorder has not been uniformly embraced. Concerns have been raised that the label of ‘disorder’ pathologizes normal variants of healthy sexual behavior or that excessive/problematic sexual behavior may be better explained as an extension of a pre-existing mental health disorder or poor coping strategies used to regulate negative affect states rather than a distinct psychiatric disorder [13]. There are significant differences between hypersexual behavior, Internet pornography use, and Sexual Addiction. If the subject exhibits only a strong sexual desire or a high interest in sexual behavior (such as frequent masturbation in the case of adolescents), without this causing discomfort for the subject, it will not be possible to make a diagnosis of Hypersexual Behavior Disorder or of Sexual Addiction. The use of online pornography is not a problematic behavior so long as it does not affect the person’s life, becoming their main daily activity. Hypersexual Disorder is a repetitive and intense preoccupation with sexual fantasies, impulses and behaviors, resulting in clinically significant distress or impairment in social, occupational or other important areas, which manifests itself over a period of at least six months and in a person who must be at least 18 years of age. A distinctive feature of the proposed disorder includes multiple failed attempts to control or decrease the amount of time an individual engages in sexual fantasies, impulses and behaviors in response to dysphoric moods or stressful life events [9]; at the same time, risks of physical or emotional harm to oneself and others are not included in the definition [14]. Individuals with problematic hypersexual behavior have high impulsivity, cognitive rigidity, poor judgment, impaired emotion regulation and excessive concern for sex [9]. Another extremely significant aspect is the impulse decontrol. In fact, these people are unable to control their sexual cravings. Neuroimaging studies using stop signal activity and go/no-go activity have shown that brain regions associated with response inhibition include the inferior frontal gyrus, insula, pre-supplementary motor area, cortex anterior dorsal cingulate, the parietal lobe and the subthalamic nucleus [15]. The results of studies on brain injury and transcranial magnetic stimulation indicate that the ability to control an automatic response is associated with the integrity of the right IFG and preSMA, anatomically linked to each other. This has led neurologists to assume that the decrease in inhibitory control on go/no-go tests is associated with the hypoactivation of the right IFG and preSMA and the alteration of the connection between these two brain areas. It has also been shown that signal reactivity and executive functioning, including response inhibition, influence each other synergistically in patients with addiction [16]. This indicates that individuals with stronger cue responsiveness will have greater problems with inhibitory behavior. In other words, addicts develop an attraction to addiction-related stimuli as these tend to grab their attention and, as previous studies on substance use addictions suggest, an abnormal increase in attention and motivation related to salient objects could turn addiction into a disinhibitory disorder and lead to loss of control. Sexual Addiction is a disorder characterized by invasive and obsessive sexual thoughts and fantasies, which lasts for an extended period of time, associated with loss of control over sexual behavior that causes negative consequences at work, emotional and social levels.

In contemporary society, the problem of Sexual Addiction is potentially worsening. People are continually bombarded with sexual stimuli, so developing an addiction of this type could become extremely easy. Internet sites with hard photos and videos, escort sites, applications and chats represent only a small part of what the Internet world offers [17]. As Kraus et al., [18] argued, digital technologies have permeated social, economic, political and cultural life, transforming social relations, the identities of subjects, as well as the patterns of production and consumption. Sexual practices have been profoundly influenced by these changes and have changed, giving rise to new phenomena of cyber-Sexual Addiction. The psychological literature interprets this technological change as an escalation and expansion of risk as people who, most likely, in the absence of hyper-stimulation would not have developed an addiction, find themselves being invaded by new forms of sexual imagination and perverse fantasy that threaten real-life relationships [13]. Sex addict behavioral disorders can be paraphilic or nonparaphilic, where the paraphilic refers to a sexual arousal that the subject feels towards objects, actions or situations considered unconventional [19]. The most common paraphilias are: exhibitionism, frotteurism, voyeurism. Non-paraphilic disorders, on the other hand, refer to continuous and pervasive sexual fantasies, hypersexuality, compulsive masturbation and sexual promiscuity [8]. Familiar factors such as the genetic predisposition to sensation seeking and hormonal factors, as well as environmental factors, such as childhood abuse, exposure to sexual contents, and the onset of psychiatric disorders, can give rise to Sexual Addiction. Psychiatric disorders comorbid with Sexual Addiction are personality disorders, anxiety disorders, and impulse control disorders [20]. Research is currently studying the traits present in obsessive-compulsive disorder, eating disorders and affective disorders, in particular bipolar disorder in which periods of mania alternate with periods of depression. Often in these people there are impulsive factors that aim to eliminate emptiness and boredom [20]. They experience a high neurochemical activation in the anticipatory phase with tachycardia and sweating. At the end of the sexual act, the reward circuit associated with it also ends and the same negative feelings as before—such as anxiety and depression—return, aggravated by a strong sense of guilt. Loss of control over one’s thoughts and behaviors generates anger and frustration, which can lead to natural mood disorders. The person will begin to have relationship, financial and often legal problems, and experience social and emotional isolation, which is one of the most serious consequences of Sexual Addiction [21].

According to Grubbs et al., the role of the media could be fundamental for the development and maintenance of problematic behaviors, including dysfunctional sexual behaviors [22]. As showed by Wright and collaborators [23], there is some social influence in learning new sexual practices and desires related to them. He integrated the concept of sexual scripting with research and theory from communication, media effects, observational learning, and information processing into a sexual script—the acquisition, activation, application model of sexual media socialization—and found supporting evidence [23] which this leads us to consider whether modifying the messages transmitted by the media could be one of the ways to oppose dysfunctional sexuality in favor of healthy sexual practices.

The objective of this exploratory study was to offer a contribution to the scientific literature by providing additional information on the impact that COVID-19 has had on the psychological level of people, and in particular to examine whether and how sexual behaviors tending to hyper activation and dependence were also modified. In this context, we speculate that this period gives us the unique opportunity to explore how a pandemic may or may not change sexual behavior on a large scale. The primary objective of this study was to evaluate the possible alteration or not towards Sexual Addiction in Italians and its possible underlying reasons. This research also evaluated the tendency to engage in hypersexual behavior, or not, during the period of confinement due to the COVID-19 pandemic.

## 2. Materials and Methods

### 2.1. Partecipants and Procedures

The individuals who participated in our research were adults and were recruited online to complete a self-report questionnaire in the period between April 2020 and April 2021. The questionnaires have been delivered via the main social networks. Participants were recruited on a voluntary basis, informed consent was received online before respondents answered the questionnaire, and data confidentiality was ensured according to the principles of the General data protection regulation (GDPR). For this reason, it was developed in Google Docs format and sent to participants electronically along with the researchers’ instructions and contact information. Participants were required to fill it in, completely and truthfully. The importance (both in general and in particular) of the questionnaire was also explained at the beginning of the questionnaire. In addition, participants were informed of the type and duration of the procedures used.

### 2.2. Measures

The tool used for this study is the SAST (Sexual Addiction Screening Test) [24]. This test was developed to measure excessive sexual behaviors as a behavioral addiction and comprises items derived from substance abuse disorders to measure symptoms, such as withdrawal, tolerance, or craving. Its creator, Patrick Carnes, compared Sexual Addiction with other addictions, describing the stages of its development and discussing the methods of intervention and treatment of choice for this disorder based on his research and clinical observations [17]. According to Carnes’ theory, the cycle of Sexual Addiction begins with the “core beliefs”, often unconscious, that the subject with Sexual Addiction possesses about him/herself, such as the belief that he/she is a bad and undeserving person, the belief that no one can love him/her for who he/she really is, the belief that his/her needs will never be met and that he/she must necessarily depend on someone else [24]. The SAST was composed of 25 dichotomous items, a review about the psychometric properties of the SAST showed that this instrument was appropriate in terms of its internal consistency, (α = 0.85 to 0.95) [25]. Total scores range from 0 to 25, with higher scores indicating higher Sexual Addiction. Scores of 13 or higher suggest the presence of a Sexual Addiction. Score above 6 indicates hypersexual behavior, and a total score of 13 or more on the SAST results in a 95% true positive rate for Sexual Addiction (i.e., a 5% or less chance of incorrectly identifying a person as a sexual addict [26]. Carnes found that this cutoff score best differentiated between people who self-identified as a sex addict and people who did not. SAST was designed to measure mainly the symptoms of Sexual Addiction, but also sexual concern, the signs and symptoms of poor sexual control and problems related to hyper-sexual behavior. SAST scores range from 0 to 25, and a score of 13 or greater suggests the presence of Sexual Addiction, and it is composed of four factors: 1—Loss of control; 2—Addiction symptoms; 3—Functional impairment; 4—Subject’s worry about getting caught [19]. The SAST is considered a reliable and valid screening for Sexual Addiction with cronbach’s α and Ω of the total score 0.93 in the paper-and-pencil format and 0.96 in the online version [26].

### 2.3. Statistic Analysis

ANOVA one-way was used in order to assess statistical differences in sexual behavior as assessed by SAST between participants with different sentimental situations (married or not). This research examined differences regarding the time spent using pornography, online and offline, before and after the pandemic period by using the *t*-test for paired measures, which was employed to verify if there were significant differences in the four sub-scales measured by the SAST tool before and after the pandemic. Descriptive statistics have also been calculated.

Information on gender, age, and level of education have been collected. The total daily time (in minutes) participants spent on the Internet at pornographic sites before and after the COVID-19 outbreak was examined. This allowed having both current and pre-pandemic data available in order to assess and define whether the time for pornographic material, both online and not, has increased or not during the pandemic period. Additionally, the risk factors for increased time spent on the Internet, as well as the severity of Internet addiction before and after the COVID-19 outbreak were also investigated.

## 3. Results

A total of 1706 survey participants have been recruited and 305 questionnaires with incomplete answers have been excluded, leaving 1401 participants for final analysis

The reference sample is made up of 671 males (47.8%) and 730 females (52.0%), for a total of 1401 people (100%), 582 (41.5%) were unmarried boys or men, 581 (41.4%) were unmarried girls or women and 50 married (3.6%) subjects for a total of 1213 (86.4 %) subjects who answered the question relating to their marital status (Table 1). Participants were asked whether or not they had love affairs, 686 subjects answered yes (48.9%), while 538 gave a negative answer (38.3%) for a total of 1224 answers (87.2 %). A total of 180 blank responses were provided (12.8%). As far as qualifications are concerned, within our sample, 65 subjects attended middle school (4.6%), 981 are first level graduates (69.9%), 202 are second level graduates (14.4%) and 153 (11.1%) did not answer concerning their education level. To better understand the population trend in relation to possible Sexual Addiction behavior, and according to SAST scoring procedure, the sample has been divided into different diagnostic categories. As seen in Table 1, 82.0% of the participants do not exhibit Sexual Addiction as assessed by SAST, 15.8% of the participants exhibit hypersexual behavior, and 2.2% show a real Sexual Addiction as assessed by SAST.

To check if there were any differences relating to the sentimental situation—single or married—(independent variables) and the factor addiction symptom and hide scores (dependent variables) we proceeded with the ANOVA one way.

Table 2 shows a significant difference in the “loss of control” and “addiction symptoms” category, in this case, a single man has significantly higher scores than the other categories. By contrast, single women have significantly higher scores than the other categories in the “functional impairment” and “hide scores” (subject’s concern of being discovered). We carried out the same analyses, this time taking into consideration the educational qualification, for which no significant difference emerged for the different categories (loss of control, addictive symptoms, functional impairment, hide score). We wanted to see if there were any significant differences with respect to the time spent using pornographic material, both online and not (dependent variable), before and after the pandemic (independent variable). To do this we proceeded with a *t*-test for paired measures.

The Table 3 above describes the average time our sample spent looking porn sites pre- and post-pandemic. In this case the *t* is similar to, 949 (d: 0.4) and the significance is equal to, 343; therefore, there are no significant differences in the use of porn material.

To conclude, we wanted to verify if there were significant differences in the four sub-scales measured by the SAST tool concerning the use of pornographic material before and after the pandemic. To do this, we proceeded with a Paired *t*-test.

Table 4 shows that the loss of control in the pre-pandemic phase has a greater weight than in the post-pandemic phase, and any statistical differences for the other variables.

## 4. Discussion

The pandemic caused by COVID-19 has been the most striking social phenomenon in recent years and has led to the emergence of clinically significant distress from a psychological point of view [27], including to the most vulnerable populations, which include those suffering from addiction. Several studies have been conducted to monitor the trend of addictions during the COVID-19 pandemic. Some studies have shown that behavioral addictions have undergone a significant increase: for example, the use of the Internet and in particular of websites relating to pornography and video games has significantly increased [28,29]. This research was conducted with the aim of investigating the aforementioned possible correlation and assessing whether dysfunctional behaviors, such as compulsive behaviors, can lead to real Sexual Addiction. Although even today Sexual Addiction has not been recognized by official nosographs, in the literature there are different definitions to explain the clinical manifestations that characterize this phenomenon still under study. As with substance use addictions, and with regard to behavioral addictions, the most accredited etiological hypothesis is the multifactorial one with neurobiological, psychological and social factors that together determine the onset, as well as the maintenance of an addiction disorder. This study showed that 82% of the participants do not demonstrate a Sexual Addiction as assessed by SAST, with 15.8% of the participants exhibiting hypersexual behavior and 2.2% showing a real Sexual Addiction as assessed by SAST. A significant difference was found in the “addiction symptoms” and “loss of control” categories only for single men, while single women have significantly higher scores in the “hide score” (subject’s worry about getting caught) and the “functional impairment” category. Moreover, among the four categories, in the pre-pandemic phase, only the loss of control it had a greater weight than in the post-pandemic phase. Finally, emerged that there are no significant differences in time spent using pornographic material (both online and not), before and after the pandemic.

The results obtained from our study can be compared with a cross-sectional survey conducted on 6003 Italian adults aged between 18- and 74-years, representative of the general Italian population [30]. The study subjects were recruited at the time of the national order to stay at home (27 April to 3 May 2020). The characteristics associated with the decrease in the frequency of sex during the lockdown were identified, differentiating between cohabiting and non-cohabiting subjects. Over a third (35.3%) of Italians reported having changed their sexual activity during the lockdown (8.4% increased and 26.9% decreased). A second study conducted by Grubbs et al. [31] states that due to the increase in time spent at home a consequent reported increase in traffic to specific pornographic websites led the authors to reflect on speculation that pornography use could generally increase over the course of the pandemic and that problematic use could also increase. To test these speculations and quantify the effects of the pandemic and the associated restrictions on social behaviors on pornography use, data from a longitudinal sample of American adults were analyzed. The present study can be observed in parallel with the results obtained from our research as the results of the same state that among those who reported use in May 2020, only 14% reported increases in use since the beginning of the pandemic and their use returned to similar levels to all other users by August 2020. In general, pornography use has had a downward trend during the pandemic, for both men and women. Problematic pornography use has had a down-ward trend for men and has remained low and unchanged in women. Collectively, these findings suggest that many concerns about pornography use during pandemic-related lockdowns were largely not supported by the available data.

Despite the relatively large sample of 1401 participants, the present study focused on limited geographical areas, leaving out the non-urban population entirely. This represents a limit of the present study, which will be addressed in a subsequent investigation. A second limit could be related to the method of administration of the questionnaire, because the online administration could have compromised the response due to disturbing factors or elements related to the processes of social desirability, which could not be controlled. A third limit is that possible alterations towards Sexual Addiction and/or the tendency to engage in hypersexual behavior during the lock-down period was assessed once but no one could predict what would happen with COVID-19.

## 5. Conclusions

The present study shows that overall, there was no increase in Sexual Addiction and hypersexual behavior during the lockdown period caused by the COVID-19 pandemic. However, we found that single men display more symptoms of Sexual Addiction than the other categories. The correlation between single men and the compulsive use of online pornography is widely discussed in the literature [32,33]. However, the literature suggests it is often associated with several medical and psychopathological causes. Studies have been performed on men suffering from erectile dysfunction, showing a correlation between increasing consumption of online pornography and erectile dysfunction [34]. In another study, Niazof et al. indicate that males with ADHD and anxious attachment show extensive use of online pornography [29]. Data on the correlation between online pornography use in men and stress caused by the pandemic are currently few, and further research is needed. Furthermore, this study showed that single women have high scores in the “hide score” category, which indicates the fear that others may discover symptoms of Sexual Addiction. Problematic sexual activity online often occurs in secret and as a lonely activity hidden from family members and, according to Levi et. al, there has been an increase in online pornography use by women in recent years [30]. It is possible that in the modern world and with the growing strength of the feminist movement, women adopt strategies that were traditionally considered masculine traits such as being assertive, risk-taking and impulsivity [35]. Results from the study by Kolmenac and Hochleitner suggest an association between frequent consumption of pornography by women and increased sexual functioning [32]. The subjects’ fear of losing control was greater in the lockdown period than in the post-lockdown period. The trauma of the COVID pandemic caused the collapse of habitual certainties in people, as well as anxiety and depression with consequent emotional dysregulation [36]. As we know, loss of control is strongly correlated to these psychopathologies; this could explain the study results regarding the increase of this factor in the period of the lockdown and its consequent decrease when the restrictions were relaxed. Based on the statistics provided by Pornhub regarding the number of users who used online pornography during the COVID-19 pandemic, we realize that the results of a general non-increase in symptoms of Sexual Addiction may not fully reflect reality and have been skewed by the phenomenon of social desirability. In our sample this is highlighted by the scores in the “hide score” category, which relates to the subject’s fear of being discovered. These data highlight how, in a historical moment such as this, we are witnessing the increased liberation of sexual mores and there is increased talk of liquid sexuality, this is still a taboo, despite the fact that the questionnaire was completed online and totally anonymous. Apparently, it would seem easy to understand that people seek to hide their potential Sexual Addiction or hypersexual behaviours from others, but also that people do not want to confess it to themselves, as it would reduce their attraction, curiosity, and often even their need. Clearly, the implementation of behaviors related to the satisfaction of sexual needs does not necessarily imply that the subject develops an addiction of this type. However, some categories of particularly vulnerable subjects could develop hypersexual behaviors and consequently a real Sexual Addiction. A man or a woman vulnerably linked to sexuality is not easy to deal with, especially when the subject realizes that he/she has lost control of himself. Shame and guilt often prevent subjects from talking about this problem and so individuals would rather deny that they have a problem than refer it to a specialist.

Further research is needed that will allow us to better understand this phenomenon in women, in disadvantaged groups, in people of different sexual orientations and in people with physical and intellectual disabilities. Furthermore, it is necessary to build an ad hoc instrument similar to the one we used, but which also contains sub-scales of the “lie” type able to bring out the incongruent answers and complete the assessment at different time points.

## Figures and Tables

**Table 1:** Participants’ characteristics.

Socio-Demographic Characteristics at Baseline	*n* (%)
Gender	
Male	671 (47.8)
Female	730 (52)
Age: mean (SD)	23.1 (5.8)
Age range	
18–24	1079 (77.1)
25–44	300 (21.4)
45–65	22 (1.5)
Marital status	*n* (%)
Unmarried Men (Celibate)	582 (41.5)
Unmarried Women (Maiden)	581 (41.4)
Married	50 (3.5)
No answer	188 (13.4)
Presence of Intimate relationship/s	
Yes	686 (48.9)
No	538 (38.3)
No answer	180 (12.8)
Education: *n* (%)	
Middle school	65 (4.6)
First level graduates	981 (69.9.)
Second level graduates	202 (14.4)
No answer	153 (11.1)
Ethnicity	
White Caucasian	1401 (100)
SAST Categories	
No Sexual Addiction	1150 (82.1)
Hypersexual Behaviour	220 (15.7)
Sexual Addiction	31 (2.2)

**Table 2:** ANOVA one way: sentimental categories and addiction symptoms.

	Maritial Status	N	M	SD	F	P
1	celibate	582	0.950	0.972	4.759	0.001
	maiden	581	0.829	0.909		
	married	50	0.620	0.725		
2	celibate	582	0.948	1.013	13.72	0.001
	maiden	581	0.700	0.846		
	married	50	0.480	0.735		
3	celibate	582	0.800	2.271	6.243	0.01
	maiden	581	1.394	3.436		
	married	50	0.840	2.794		
4	celibate	582	0.606	0.768	13.62	0.001
	maiden	581	0.826	0.823		
	married	50	0.460	0.734		

Legend: 1 = Loss of control; 2 = Addiction symptoms; 3 = Functional impairment; 4 = Hide Score.

**Table 3:** Paired *t*-test related to time dedicated to the use of pornographic material, both online and not, before and after the pandemic.

	M	SD	*t*	*p*
Sex Pre-Pandemic	18.43	11.718	0.949	0.343
Sex Post-Pandemic	18.35	11.139		

**Table 4:** Paired *t*-test related to the four SAST categories before (B) and after (A) the pandemic.

Categories	M	SD	*t*	*p*
B Loss of control	1.020	1.00	8.08	0.001
A Loss of control	0.895	0.942		
B Addiction symptoms	0.8104	0.921	7.11	1.21
A Addiction symptoms	0.8821	0.932		
B Functional impairment	1.023	2.91	7.85	0.98
A Functional impairment	1.112	2.36		
B Hide score	0.796	0.801	8.32	0.62
A Hide score	0.803	0.851		

## Data Availability

The data presented in this study are available on request from the corresponding author. The data are not publicly available due to privacy.
